# ﻿*Carrierealeyensis*, a new species of Salicaceae from limestone areas of Guangxi, China

**DOI:** 10.3897/phytokeys.248.129824

**Published:** 2024-11-05

**Authors:** Zhao-Cen Lu, Zhi-Rong Liu, Ming-Lin Mo, Shi-Li Chang, Wei-Bin Xu

**Affiliations:** 1 Guangxi Key Laboratory of Plant Conservation and Restoration Ecology in Karst Terrain, Guangxi Institute of Botany, Guangxi Zhuang Autonomous Region and Chinese Academy of Sciences, Guilin, 541006, Guangxi, China Guangxi Zhuang Autonomous Region and Chinese Academy of Sciences Guilin China; 2 Nonggang Karst Ecosystem Observation and Research Station of Guangxi, Chongzuo, 532499, Guangxi, China Nonggang Karst Ecosystem Observation and Research Station of Guangxi Chongzuo China; 3 Guangxi Forestry Inventory and Planning Institute, Nanning, 530011, Guangxi, China Guangxi Forestry Inventory and Planning Institute Nanning China; 4 College of Life Sciences, Guangxi Normal University, Guilin, 541006, Guangxi, China Guangxi Normal University Guilin China

**Keywords:** *Carriereacalycina*, *Carriereadunniana*, morphology, new taxa, taxonomy

## Abstract

*Carrierealeyensis* Z.C.Lu & W.B.Xu, a new species of Salicaceae was discovered from limestone areas of Guangxi, China. The morphology of *C.leyensis* is similar to *C.dunniana*, but differs by its evergreen nature; shorter petioles, only 3–8 mm long, and tomentose or glabrous when old; elliptic leaf blade with cuneate base; shorter inflorescence (1.8–4.5 cm long); smaller flowers; and smaller capsules (1.7–2.7 cm long, 5–9 mm in diam.).

## ﻿Introduction

*Carrierea*Franchet (1896: 498) is a small genus in Salicaceae, distributed from southern China to northern Vietnam (Lai 1999; Yang and Zmarzty 2007). *Carrierea* is closely related to *Poliothyrsis* Oliv. and *Itoa* Hemsl., all of which have ellipsoidal to spindle-shaped capsules that split from both the base and the apex (Alford 2005). These genera were once placed in the Flacourtiaceae (Fan 1990, 1995; Yang and Zmarzty 2007), but molecular (Chase et al. 2002) or molecular and morphological data (Alford 2005) indicate that these genera are very closely related to *Salix* L. and *Populus* L. (Salicaceae*s. str.*). Four names have been published in *Carrierea* previously, but recent revisions have treated *C.rehderiana* Sleumer as a synonym of *C.calycina* Franch. (1896: 498), and *C.vieillardii* Gagnep. as a synonym of *Itoaorientalis* Hemsl. (Lai 1999; Yang and Zmarzty 2007). *Carriereacalycina* and *C.dunniana*Léveillé (1911: 458) are accepted taxa, both of which are distributed in China, and *C.calycina* is endemic to China (Yang and Zmarzty 2007).

During our investigation of plant diversity from July 2023 to May 2024, an unusual plant that could belong to an unknown species of Salicaceae with flowers and mature capsules was collected from limestone forests in Leye County, Baise City, Guangxi, China. Based on the smaller capsule, the previous year’s zigzag dehiscence capsule and the shorter inflorescence with 2–11 flowers, this unknown species was placed in the genus *Carrierea*. After carefully checking the morphological characters of the specimens, consulting relevant literature (Fan 1990, 1991, 1995; Lai 1999; Yang and Zmarzty 2007), studying herbarium specimens, and examining the other related specimens of *Carrierea*, we confirmed that this species is new to science, and it is described below.

## ﻿Materials and methods

Specimens of this new species were observed, photographed, and collected from Leye County, Baise City, Guangxi, China. Herbarium specimens were deposited at CSH, GXMG, GXMI, IBK, IBSC, KUN and PE (Herbarium codes follow Thiers 2022). Morphological characters of the specimens, including the size, shape, and color of bark, petioles, leaf blades, bracts, bracteoles, inflorescences, staminate and pistillate flowers, sepals, capsules and seeds were recorded. Online images of the other specimens of *Carrierea* were examined from the Chinese Virtual Herbarium (https://www.cvh.ac.cn/), JSTOR Global Plants (https://plants.jstor.org), and Kew Herbarium Catalogue (http://apps.kew.org/herbcat/gotoHomePage.do). The morphological description for the new species is based on the cited type specimens (holotype, isotypes, and paratypes).

## ﻿Taxonomic treatment

### ﻿ Carrierea leyensis

Taxon classificationPlantaeMalpighialesSalicaceae

Z.C.Lu & W.B.Xu sp. nov.

CF54AF72-553E-5951-803E-AD7085BF456A

urn:lsid:ipni.org:names:77351405-1

Figs 1 , 2 , 3

#### Diagnosis.

*Carrierealeyensis* Z.C.Lu & W.B.Xu differs from *C.dunniana* H.Lév. in its evergreen nature; shorter petioles, only 3–8 mm long, and tomentose or glabrous when old; elliptic leaf blades with cuneate base; shorter inflorescences; smaller flowers; and smaller capsules.

**Figure 1.**
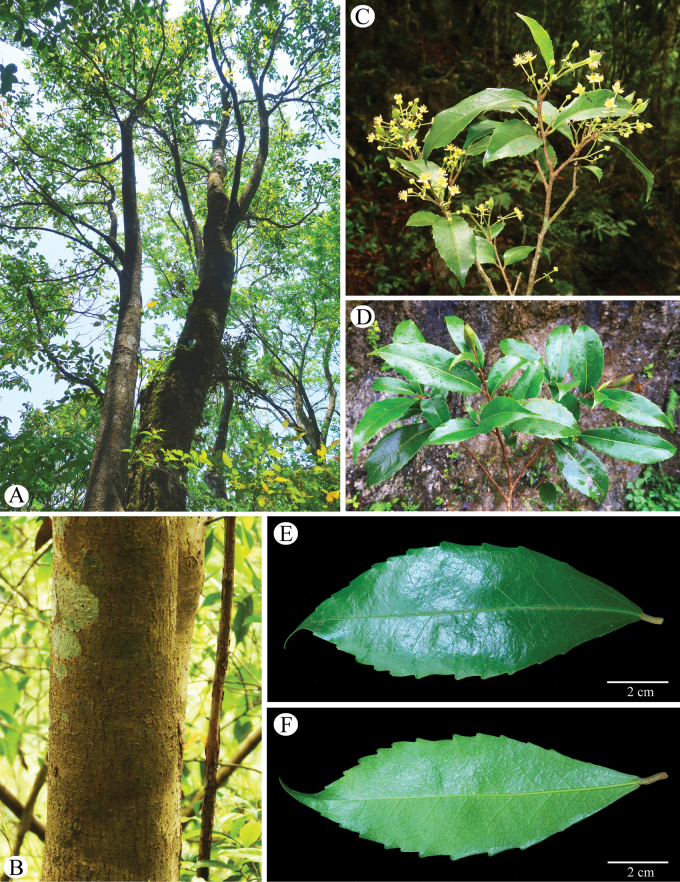
*Carrierealeyensis* sp. nov. **A** habit **B** trunk **C** flowering branches **D** fruiting branches **E** leaf, view from adaxial side **F** leaf, view from abaxial side.

#### Type.

China • Guangxi: Baise City, Leye County, Tongle Township, Fengdong Village, Laomudong, around the point 106.482109°E, 24.75169°N, in forests of limestone slope, elevation ca. 1330 m, 4 May 2024, *W. B. Xu, Z. C. Lu, M. L. Mo, S. L. Chang & J. Q. Huang 18270* (holotype: IBK00461833; isotypes: IBK00461831, IBK00461832).

**Figure 2.**
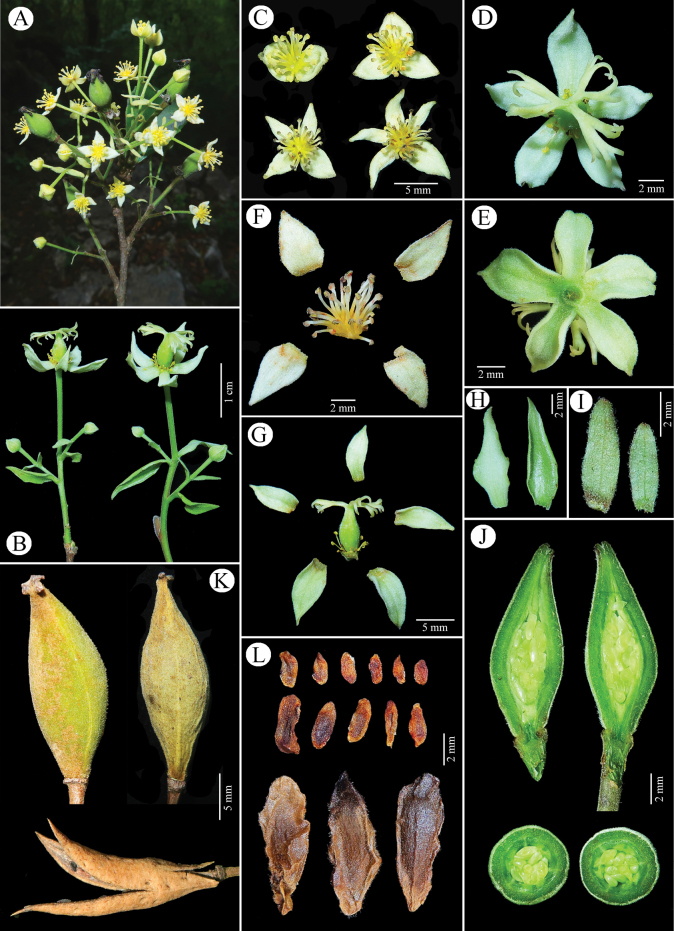
*Carrierealeyensis* sp. nov. **A** flowering branches with staminate flowers and young capsules **B** inflorescences **C** staminate flowers in frontal view **D** pistillate flower in frontal view **E** pistillate flower in dorsal view **F** dissection of staminate flower **G** dissection of pistillate flower **H** bracts **I** bracteoles **J** long-section and cross-section of ovary **K** capsules **L** seeds.

#### Description.

Trees or small trees, monoecious, evergreen, 5–12 m tall; bark gray-brown; branchlets grayish, with white lenticels and leaf marks, tomentose, glabrous when old; winter buds conical, scales hairy; stipules absent. Petiole 3–8 mm long, tomentose to glabrous when old; leaf blade greenish abaxially, deep green adaxially, glabrous, elliptic, (2.8–)4–9.5(–12.5) × 1.7–4.6 cm, leathery or thinly leathery, both surfaces glabrous or abaxially sparsely appressed-villous along midveins, weakly 3-veined at base, lateral veins 5–8 pairs, veins distinct on both sides, midvein raised below, base cuneate, margin remotely serrate, with spheroidal to torus-shaped glands at the tips of the teeth (salicoid teeth), apex acuminate to long acuminate. Inflorescence terminal or axillary, 2–11-flowered, rarely single flower axillary, 1.8–4.5 cm long including flowers, tomentose, pistillate flowers in terminal part of inflorescence, staminate ones in lower part; bracts ovate-lanceolate, 1–1.35 cm long, papery, both surfaces sparsely to densely tomentose. Pedicels 0.5–2.5 cm long, 2-bracteolate near middle; bracteoles similar to bracts, opposite, narrowly oblong, 3–7 mm long, papery, both surfaces sparsely to densely tomentose. Sepals (2 or 3 rare) 4–5, valvate, ovate-deltoid to oblong, 3.5–8 × 2–3 mm, papery, both sides tomentose, apex acute. Petals absent. Staminate flowers: smaller than pistillate flowers, stamens with filaments unequal in length, 2–3 mm long, glabrous; anthers ca. 0.5 mm long. Pistillate flowers: staminodes like stamens but much reduced; ovary superior, elliptic, densely tomentose, 0.6–1 cm long; placentas 3 or 4; styles 3 or 4, 0.5–1 mm long, connate at least at base to form a column, sparsely tomentose like the ovary; stigmas reflexed, drying black, flattened, triangular, 2–3 mm long, irregularly lobed, glabrous. Capsule fusiform, slightly curved, 1.7–2.7 cm long, 5–9 mm in diam., tomentose, valves splitting from both apex and base; fruiting pedicel stout, 1–2.8 cm long; seeds compressed, including wing 9–12 mm long; sterile seeds smaller.

**Figure 3.**
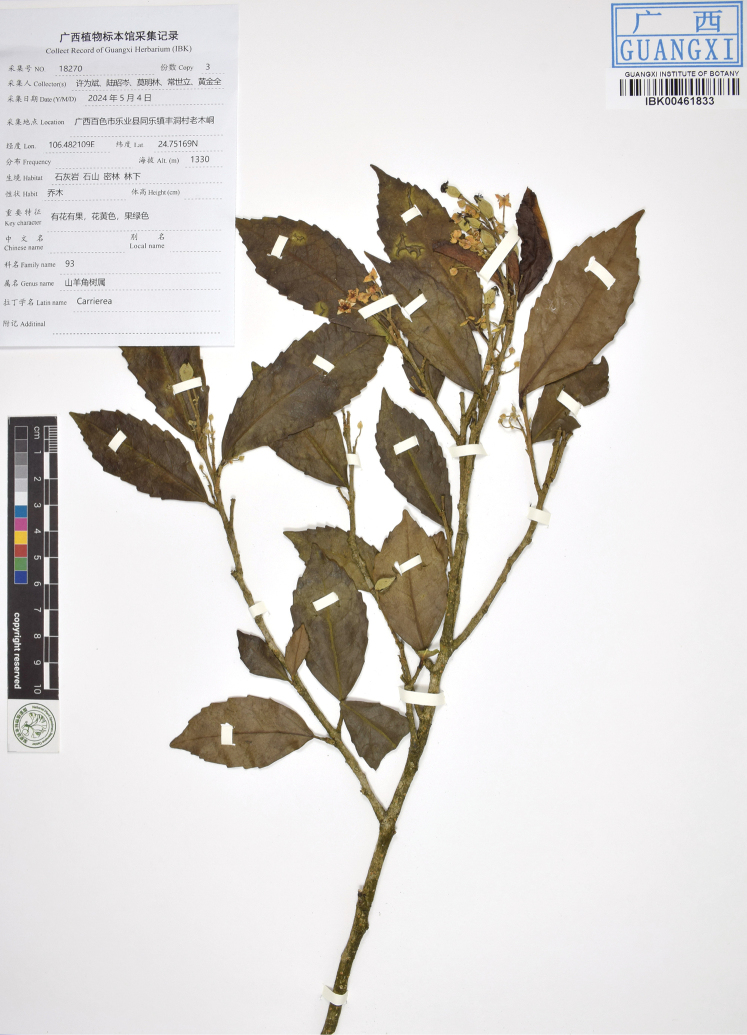
The holotype sheet of *Carrierealeyensis* (IBK).

#### Etymology.

The specific epithet ‘*leyensis*’ refers to the locality where the new species was collected.

#### Phenology.

Flowering April to May (spring); fruiting July to October (summer to autumn).

#### Distribution and habitat.

*Carrierealeyensis* has only been collected from limestone areas of Leye County, Baise City, Guangxi, China. It grows sporadically in forests on limestone slopes, at an elevation of 1100–1350 m. Associated species include *Handeliodendronbodinieri* (H.Lév.) Rehder, *Pistaciachinensis* Bunge, *Pittosporumtonkinense* Gagnep., *Machiluscavaleriei* H.Lév., *Triadicarotundifolia* (Hemsl.) Esser, *Jasminumlanceolaria* Roxb., *Eriobotryaseguinii* (H.Lév.) Cardot ex Guillaumin, *Carexbrunnea* Thunb., *Ophiorrhiza* species, and *Carpinus* species.

#### Conservation status.

The new species has been found in three localities in Leye County, Baise City, Guangxi, China. We did not complete additional surveys in the area, so the number of living populations is unknown. According to the IUCN Red List Categories and Criteria (IUCN Standards and Petitions Committee 2022), *Carrierealeyensis* should be considered in the Data Deficient (DD) category at present.

#### Additional specimens examined

**(paratypes).** China • Guangxi: Baise City, Leye County, Tongle Township, Fengdong Village, around the point 106.502383°E, 24.769041°N, limestone slope, alt. 1190 m, 30 July 2023, *W. B. Xu, Z. C. Lu, M. L. Mo, S. L. Chang & J. Q. Huang 16830* (IBK, GXMG, IBSC); • ibid., 30 July 2023, *W. B. Xu, Z. C. Lu, M. L. Mo, S. L. Chang & J. Q. Huang 16832* (IBK, PE, KUN); • ibid., 10 October 2023, *W. B. Xu, Z. C. Lu, S. L. Chang & J. Q. Huang 17742* (IBK, CSH); • Tongle Township, Longmen Village, Caojiadongtun, around the point 106.456363°E, 24.736441°N, limestone slope, alt. 1230 m, 31 December 2023, *W. B. Xu, Z. C. Lu, S. L. Chang & J. Q. Huang LZC2195* (IBK, GXMI); • ibid., 24 September 2023, *Z. R. Liu LKY-YC468* (IBK); • ibid., 4 May 2024, *W. B. Xu, Z. C. Lu, M. L. Mo, S. L. Chang & J. Q. Huang 18273* (IBK, CSH); • ibid., 4 May 2024, *W. B. Xu, Z. C. Lu, M. L. Mo, S. L. Chang & J. Q. Huang 18274* (IBK, GXMI); • Tongle Township, Fengdong Village, Laomudong, around the point 106.481868°E, 24.751426°N, limestone slope, alt. 1300 m, 4 May 2024, *W. B. Xu, Z. C. Lu, M. L. Mo, S. L. Chang & J. Q. Huang 18271* (IBK, PE, KUN); • ibid., 4 May 2024, *W. B. Xu, Z. C. Lu, M. L. Mo, S. L. Chang & J. Q. Huang 18264* (IBK, GXMG, IBSC); • ibid., 4 May 2024, *W. B. Xu, Z. C. Lu, M. L. Mo, S. L. Chang & J. Q. Huang 18266* (IBK).

#### Notes.

**Figure 4.**
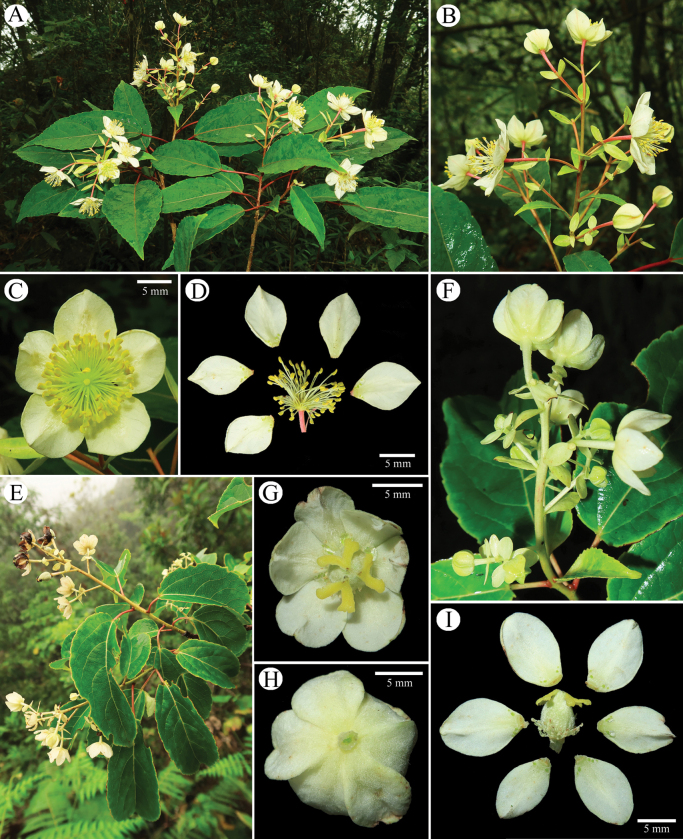
*Carriereadunniana***A** flowering branches **B** inflorescence **C** staminate flower in frontal view **D** dissection of staminate flower; *Carriereacalycina***E** flowering branches **F** inflorescence **G** pistillate flower in frontal view **H** pistillate flower in dorsal view **I** dissection of pistillate flower.

*Carrierealeyensis* can be easily distinguished from the other two species of *Carrierea* in its evergreen nature, shorter petioles, the shape of leaf blades, shorter inflorescences, smaller flowers, and smaller capsules. The morphological differences between *C.leyensis* and the two related species *C.dunniana* (Fig. 4A–D) and *C.calycina* (Fig. 4E–I) are summarized in Table 1.

**Table 1.** Comparison among *Carrierealeyensis*, *C.calycina* and *C.dunniana*.

Characters	*C.leyensis*	*C.calycina*	*C.dunniana*
Habit	Evergreen	Deciduous	Deciduous
Petiole	3–8 mm long, tomentose or glabrous when old	(2.5) 3–7 cm long, pubescent or glabrous	2–5 cm long, glabrous
Leaf blade	Elliptic, base cuneate, apex acuminate to long acuminate	Ovate-oblong, oblong, or slightly obovate, less often elliptic, base rounded to cordate, apex obtuse to acute	Ovate to oblong, base rounded, apex acuminate
Inflorescence	Terminal or axillary, 1.8–4.5 cm long	Terminal, 5–10 cm long	Terminal or axillary, 7–15 cm long
Sepals	(2 or 3 rare) 4–5, ovate-deltoid to oblong, 3.5–8 mm long	4–6, broadly ovate, 1.5–2 cm long	4–5, obovate to elliptic, 5–10 mm long
Bracts	Ovate-lanceolate, 1–1.35 cm long	Lanceolate to narrowly elliptic, 1–3 cm long	Ovate, 5–10 mm long
Bracteoles	Narrowly oblong, 3–7 mm long	Narrowly oblong, 4–8 mm long	Broadly oblong, ovate, or elliptic, 2.5–5 mm long
Capsule	1.7–2.7 cm long	3–8 cm long	2.5–4 cm long

This study also provides field documentation that individuals of *Carrierea* can be monoecious. Although some reports of *Carrierea* indicate that it is dioecious, our results affirm the observations of Yang and Zmarzty (2007), who noted that the type specimens of *C.dunniana* and *C.calycina* had both pistillate and staminate flowers.

### ﻿Identification key to the species of *Carrierea*

**Table:** 

1	Petiole 3–8 mm long; leaf blade elliptic, base cuneate	***C.leyensis***
–	Petiole 2–7 cm long; leaf blade ovate-oblong, oblong, ovate or slightly obovate, base rounded or cordate	**2**
2	Sepals 1.5–2 cm long, base cordate; bracts 10–30 mm long, bracteoles 4–8 mm long; capsule 3–7 cm long	***C.calycina***
–	Sepals 0.5–1 cm long, base cuneate or only slightly cordate; bracts 5–10 mm long, bracteoles 2.5–5 mm long; capsule 2.5–4 cm long	***C.dunniana***

## Supplementary Material

XML Treatment for Carrierea leyensis

## ﻿Additional information

### Author ORCIDs

Zhao-Cen Lu https://orcid.org/0000-0002-1725-0247

Zhi-Rong Liu https://orcid.org/0009-0001-5171-5693

Ming-Lin Mo https://orcid.org/0009-0000-8895-5474

Shi-Li Chang https://orcid.org/0009-0005-9801-4121

Wei-Bin Xu https://orcid.org/0000-0002-5602-8753

### Data availability

All of the data that support the findings of this study are available in the main text.
